# Phytochemical Composition and Toxicological Screening of Anise Myrtle and Lemon Myrtle Using Zebrafish Larvae

**DOI:** 10.3390/antiox13080977

**Published:** 2024-08-12

**Authors:** Paolin Rocio Cáceres-Vélez, Akhtar Ali, Alexandre Fournier-Level, Frank R. Dunshea, Patricia Regina Jusuf

**Affiliations:** 1 School of Biosciences, The University of Melbourne, Parkville, VIC 3010, Australia; alexandre.fournier@unimelb.edu.au; 2 School of Agriculture, Food and Ecosystem Sciences, The University of Melbourne, Parkville, VIC 3010, Australia; akali@student.unimelb.edu.au (A.A.); fdunshea@unimelb.edu.au (F.R.D.); 3 Faculty of Biological Sciences, The University of Leeds, Leeds LS2 9JT, UK

**Keywords:** phytochemical characterization, antioxidant capacity, native Australian plants, anise myrtle, lemon myrtle, toxicological screening, zebrafish

## Abstract

Plants are an immense source of drugs, and 50% of modern pharmacopeia has a plant origin. With increasing life expectancy in humans, many age-related degenerative diseases converge on oxidative cellular stress pathways. This provides an opportunity to develop broad treatments by targeting the cause of common pathologic cell degeneration. Toxicological effects can be readily assessed in a live animal model system to establish potential fauna for clinical use. Here, we characterized and evaluated the antioxidant potential and toxicological effects of anise myrtle (*Syzygium anisatum*) and lemon myrtle (*Backhousia citriodora*) leaves. Using zebrafish larvae, a model for high-throughput pre-clinical in vivo toxicology screening, we identified safe levels of extract exposures for development of future therapeutics. The antioxidant capacity and toxicity were very similar in these two myrtles. The LC_50_-96h for anise myrtle was 284 mg/L, and for lemon myrtle, it was 270 mg/L. These measurements are comparable to ongoing studies we are performing using the same criteria in zebrafish, which allow for robust testing and prioritization of natural fauna for drug development.

## 1. Introduction

As human life expectancy increases, age-related diseases driven by cellular oxidative stress are increasing at alarming rates [1]. Despite an abundance of counteracting antioxidants, the lack of effective therapies highlights knowledge gaps limiting the progress of clinical translation. Limitations include the lack of standardized platforms to compare the effectiveness and safety of different antioxidant classes in vertebrates. Additionally, once antioxidants have been identified, drug delivery methods still need to be developed to optimise formation, bio distribution and systemic bioavailability. Botanically derived bioactive antioxidants can counteract oxidative stress-induced cellular damage [2,3]. As antioxidants come in various subclasses, direct identification and efficacy assessment of novel compounds using a high throughput in vivo testing platform is critical for streamlining the testing of promising drugs. Here, we present the first study directly analyzing bioactivity in conjunction with a pre-clinical live vertebrate toxicology screening method for each sample. This allows for direct cross-correlation using the same sample, as extraction methods and storage can significantly change composition and thus toxicity.

Australia’s megadiversity offers a vast number of plants with distinct mixtures of antioxidant compounds [4,5,6], which can be potentially used to develop treatments for oxidative stress-related diseases with enhanced safety characteristics and competitive performance [7,8,9]. Our study focused on two native Australian plants, anise myrtle (*Syzygium anisatum*) and lemon myrtle (*Backhousia citriodora*). Anise and lemon myrtle belong to the Myrtaceae family, which includes eucalypt trees [10]. Anise myrtle, or ringwood, is characterised by its undulate leaves that emit a characteristic aniseed fragrance. It features creamy white flowers similar to those of the eucalyptus and tiny, dry fruiting bodies resembling capsules. Anise myrtle is a tall tree that may grow up to 50 m in height and is mostly found in Bellinger and Nambucca, two small valleys in northeastern New South Wales [11].

Native Australians have used anise and lemon myrtle both for cooking and in healing [12]. Both plants are rich in antioxidants [13,14], though the yield and retention of their phytochemicals depend critically on the extraction method, as described by Saifullah and coworkers in 2023 [15]. As flavourings, their leaves have been used in and on various culinary products such as shortbread, milk-based dishes, pasta, macadamia nuts, and vegetable oils [12,16]. Both myrtles have also been used in fragrances, personal care goods, and as flavour enhancers in European cuisine and phytotherapy [9]. The leaves are frequently used in the form of dehydrated flakes or as a concentrated flavour extract that is enclosed in capsules to prolong its shelf life. Additionally, the functional properties of lemon and anise myrtle have been attributed to their essential oils [10,11]. Lemon myrtle extracts possess anti-inflammatory benefits [17]. Insect repellents and antiviral treatments, such as for *Molluscum contagiosum*, also use them [18]. Thus, they have recently sparked interest as effective anti-fungal chemicals and food preservatives [12,19,20].

Considering the bioactive potential of these plants, performing a toxicology screening for drug discovery is necessary. Thus, toxicological characterization of anise myrtle and lemon myrtle must be first completed to identify safe concentrations and minimize potential adverse effects for subsequent efficacy testing [21]. Importantly, sample-to-sample variation due to extraction methods means that direct correlation in the same sample is critical to understanding compounds’ benefit–welfare ratio, which might be considered for future translational use. To address this, we have used a rapid, non-invasive, in vivo screening system using zebrafish embryos as a preclinical model [22,23], which represents an essential bridge between in vitro assays and mammalian studies [24,25]. Zebrafish possess features particularly suitable for screening of therapeutic compounds [26,27,28]. Zebrafish embryos are widely used to undertake unbiased screening of compounds by directly correlating toxicity and efficacy and identifying the most promising candidate molecules [26,29,30]. Vertebrate organisms are powerful models and useful screening platforms for studying human disease due to their high genetic, anatomical, and neural conservation with humans and ease of manipulation [31,32]. This work undertakes a toxicological screening of native plant extracts, namely anise and lemon myrtles, to determine safe concentrations of novel therapeutic compounds for further clinical applications. The outcomes of our previous studies [26,27] form the basis for an understanding of how many botanical extracts that are deemed beneficial might represent synergistic mixtures of antioxidants to be considered for treatment development.

## 2. Materials and Methods

### 2.1. Material and Chemicals

All chemicals were at least analytical, HPLC, and LC-MS-grade and purchased from Sigma Aldrich. Gallic acid, Folin–Ciocalteu’s phenol reagent, sodium carbonate, DPPH, ABTS salt, LC-MS-grade formic acid, Trolox, potassium ferricyanide, trichloroacetic acid, FeCl_3_, NaCl, KCl, CaCl_2_, and MgSO_4_ were purchased from Sigma Aldrich (Castle Hill, NSW, Australia). HPLC vials and syringe filters were purchased from Agilent Technologies. Ethanol, methanol, and 96-well plates were purchased from Thermo Fisher Scientific (Scoresby, VIC, Australia).

### 2.2. Extraction of Phytochemicals

The extracts were prepared as described in Cáceres-Vélez et al. [26]. Briefly, one gram of fresh leaves of each anise myrtle (*Syzygium anisatum*) and lemon myrtle (*Backhousia citriodora*), collected from Peppermint Ridge Farm (Tyrnong North, VIC, Australia), was blended independently in 10 mL of 70% analytical grade ethanol and incubated at 4 °C at 120 rpm for 24 h. Then, the extracts were filtered, and the solvent was eluted by lyophilization.

### 2.3. Measurement of Total Phenols and Antioxidant Potential

#### 2.3.1. Total Phenolic Content (TPC)

The extracts’ total phenolic content was analysed using a modified version of the spectrophotometric approach reported by Kiani et al. [33]. In total, 25 µL of extracts, 25 µL of Folin–Ciocalteu reagent solution (diluted 1:3 with water), and 200 µL of water were combined and placed in a 96-well plate from Corning Inc., located in Midland, NC, USA. The mixture was then incubated at 25 °C for 5 min. After adding 25 µL of 10% (*w*/*w*) Na_2_CO_3_, a further incubation period of 1 h at 25 °C was required. Subsequently, the spectrophotometer plate reader was used to detect the absorbance at a wavelength of 765 nm. A calibration curve was created using a gallic acid standard with concentrations ranging from 0 to 200 µg/mL to measure the total phenolic content. The findings are reported in milligrams of gallic acid equivalents per gram.

#### 2.3.2. 2,2-Diphenyl-1-picrylhydrazyl (DPPH) Activity

DPPH activity was measured using the method described by Ebrahimi et al. [34] with some modifications; 25 μL of the sample extract was combined with 275 μL of 100 micromolar DPPH in methanol to determine the radical scavenging activity. The mixture was then put into a 96-well plate and incubated at 25 °C for 30 min. The DPPH was measured by comparing with the Trolox (0–100 μg/mL) at 517 nm.

#### 2.3.3. 2,2′-Azinobis-(3-ethylbenzothiazoline-6-sulfonate) (ABTS) Activity

ABTS activity was assessed by conducting an ABTS decolorization experiment, per Ali et al. [35]. A mixture was prepared to acquire the fresh ABTS^+^ dye by combining 6.25 mL of a 7 mmol/L ABTS solution with 110 µL of a 140 mmol/L potassium persulfate solution and incubated for 16 h in dark. Moreover, high-quality ethanol was used to dilute the produced ABTS^+^ dye to achieve an absorbance of 0.70 at 734 nm. Then 290 microliters of ABTS radical cation reagent was combined with 10 microliters of the sample solution. The mixture was allowed to incubate for 6 min in a dark area at room temperature. Subsequently, the absorbance was measured at a wavelength of 734 nm, and a standard curve was constructed using a range of concentrations of Trolox (0–200 μg/mL) in methanol.

#### 2.3.4. Reducing Power Activity (RPA)

The RPA was determined by adding 10 μL of extract, 25 μL of 0.2 M phosphate buffer with a pH of 6.6, and 25 μL of 1% K_3_[Fe(CN)_6_] following the method of Ebrahimi et al. [34] with slight modifications. The mixture was then incubated at 25 °C for 20 min. Next, the reaction was halted by adding 25 μL of a 10% trichloroacetic acid solution. Then, 85 μL of water and 10 μL of FeCl_3_ were added. The mixture was then incubated for an additional 15 min at 25 °C. Subsequently, absorbance was measured at a specific wavelength of 750 nm. Trolox (0–600 μg/mL) was used as an external standard.

### 2.4. High-Performance Liquid Chromatography Mass Spectrometry (HPLC-MS) Analysis

Phytochemicals were identified following the protocol described by Ali et al. [36] using an Agilent 6520 Accurate-Mass QTOF machine. Phytochemicals were identified using MassHunter Workstation Software (version B.06.00). The trials were conducted three times. The quantification of individual chemicals was accomplished by employing the methodology described in Cáceres-Vélez et al. [26]. The detection wavelengths were configured at 280 nm, 320 nm, and 370 nm. The chemicals were measured in triplicate based on the dry weight (μg/g). Our previous study reported the standard curves of individual compounds [37].

### 2.5. Acute Toxicity Test Using Zebrafish Embryos as In Vivo Model

A fresh stock solution of each plant extract was prepared in autoclaved E3 medium (containing 5 mM NaCl, 0.17 mM KCl, 0.33 mM CaCl_2_, and 0.33 mM MgSO_4_). The stock solution was then diluted to obtain various concentrations (0, 15, 30, 60, 120, 240, and 480 mg/L) for both anise and lemon myrtle extracts. At 3 h post-fertilization (hpf), zebrafish embryos were exposed to a static system as described in 2022 by Cáceres-Vélez et al. [26] and Ali et al. [27]. Briefly, healthy embryos were placed individually into 46-well plates (one embryo/well) containing 500 μL/well of test solution. Phenotypic and behavioral changes were recorded daily (24, 48, 72, and 96 h of exposure) for each embryo, using a stereomicroscope (LEICA M80). The endpoints examined were mortality, developmental alterations and morbidity which encompasses coagulation, deceased embryo/larvae, heart beating, lack of blood circulation in the tail, balance disorder, delays in hatching, pigmentation, and yolk sac absorption, as well as the presence of cardiac and yolk sac oedema. Additionally, malformations in the head, eyes, spine, tail, and somites were quantified. Balance disorder was classified as a behavioural modification. The experiments were performed in triplicate (*n* = 60 embryos per concentration) for each plant extract, in accordance with the Australian code for the care and use of animals for scientific purposes and the regulations of the Animal Welfare and Animal Ethics Committee of the University of Melbourne (Melbourne, Australia).

### 2.6. Statistical Analysis

The data were analyzed by one-way analysis of variance (ANOVA) followed by Dunnett’s multiple comparisons test with a significance threshold set to 5%, and the LC_50_ was determined by fitting a Probit model to the data in GraphPad Prism v9.3.0.

## 3. Results

### 3.1. Characterization of Australian Native Plant Extracts

#### 3.1.1. Total Phenolic Content and Antioxidant Activities

Anise myrtle and lemon myrtle showed different phenolic and antioxidant profiles, as measured using TPC, DPPH, ABTS, and RPA assays (Table 1).

Lemon myrtle showed a higher TPC than anise myrtle, possibly due to a higher concentration of flavonoids, including catechin, epicatechin, (-)-epicatechin 3-*O*-gallate, procyanidin B2, and isorhamnetin in lemon myrtle. The TPC content of anise myrtle was comparable to strawberry gum (*Eucalyptus olida*) (36.57 mg GAE/g), but higher than Davidson plum (*Davidsonia pruriens*) (32.49 mg GAE/g) and lower than quandong peach (*Santalum acuminatum*) (42.85 mg GAE/g) [35]. The TPC content of lemon myrtle is comparable with Chinese star anise (*Illicium verum*) (53.89 mg GAE/g), but lower than bush mint (*Mentha saureioides*) (57.70 mg GAE/g), and higher than river mint (*Mentha australis*) (46.59 mg GAE/g) [36] and villous amomum fruit (*Wurfbaina villosa*) (46.02 mg GAE/g) while around 2 times higher than muntries (*Kunzea pomifera*). Previously, we reported the TPC of anise myrtle (55.9 mg/g) and lemon myrtle (31.4 mg/g). In another study, we measured the TPC of anise myrtle (52.49 mg/g) and lemon myrtle (28.77 mg/g) [37]. The plants’ antioxidant capacity is vital in counteracting detrimental free radicals, diminishing oxidative stress, averting chronic illnesses, and promoting general well-being and longevity. This study measured the antioxidant capacity of anise and lemon myrtles using DPPH, ABTS, and RPA. Lemon myrtle showed higher antioxidant potential than anise myrtle (Table 1) The high antioxidant activity of Australian myrtles is due to their abundant concentration of phenolic compounds, especially flavonoids. These bioactives can efficiently eliminate free radicals, enhancing their potential to avoid or decelerate the advancement of many illnesses. Previously, a lower ABTS was measured in Maria Rita myrtle leaves (135.7 ± 10.21 mM TE/g DW), Maria Antonetta (242.6 ± 10.93 mM TE/g DW), Sofia (245.03 ± 1.21 mmol TE/g DW) and *Myrtus communis* leaves, such as Giavanna (173.9 ± 3.7 mmol TE/g DW, than Australian myrtles [38]. While Konczak et al. [13] measured the FRAP of aniseed myrtle (2.2 mM TE/g DW) and lemon myrtle (1.2 mM TE/g DW), they also quantified the oxygen radical absorbance capacity (ORAC) of aniseed myrtle (2.6 mM TE/g) and lemon myrtle (3.4 mM TE/g), which are much lower than those in this study.

#### 3.1.2. HPLC-MS Screening and Quantification of Phytochemicals in Myrtles

The phytochemical composition of plant extracts was characterized and identified using LC-MS/MS (Figure S1). We identified 183 metabolites (Table S1). These metabolites were validated using fragment MS/MS spectra (Table S1). We identified 12 hydroxybenzoic acids and derivatives; 21 hydroxycinnamic acids and derivatives; 6 other phenolic acids; 15 flavanols; 22 flavonols; 25 flavones; 6 chalcones and dihydrochalcones; 8 flavanones; 8 isoflavonoids; 2 extended flavonoids; 14 other flavonoids; 10 tannins; 8 coumarins and derivatives; 2 phenolic terpenes; 2 tyrosols; 4 stilbenes; 6 lignans; 9 other polyphenols; 2 limonoids; and 1 sesquiterpenoids.

The most abundant compounds in anise and lemon myrtles were quantified/semi-quantified (Table 2). Phenolic acids were the most abundant group quantified in this study. Twenty-four phenolic acids were quantified, and epicatechin was found at a higher concentration (4656.04 ± 230.92 μg/g) in anise myrtle and catechin (5903.02 ± 762.73 μg/g) in lemon myrtle compared to other metabolites. Syringic acid, caffeic acid, caftaric acid, ellagic acid arabinoside, ellagic acid glucoside, and myricetin 3-arabinoside were only identified in anise myrtle, while 3,4-*O*-dimethygallic acid, resveratrol, sinapic acid, catechin, and epigallocatechin were only identified in lemon myrtle. Overall, flavonoids are abundant in Australian myrtles. Higher concentrations of myricetin 3-arabinoside (1.2 mg/g), ellagic acid glucoside (0.8 mg/g), isovitexin (3.5 mg/g), ellagic acid arabinoside (2.5 mg/g), syringic acid (1.2 mg/g), quercetin (0.4 mg/g) procyanidin B2 (0.4 mg/g) were measured in anise myrtle while epicatechin (3.8 mg/g), (-)-epicatechin 3-*O*-gallate (1.5 mg/g), procyanidin B2 (1.3 mg/g), isovitexin (0.4 mg/g), quercetin (0.2 mg/g), and isorhamnetin (0.2 mg/g) were measured in lemon myrtle. Previously, Rupesinghe et al. [4] also identified catechin, epicatechin, procyanidin B2, epigallocatechin, quercetin, and *p*-coumaric acid in Australian myrtles.

### 3.2. Acute Toxicity Test Using Zebrafish Embryos as In Vivo Model

**Figure 1:**
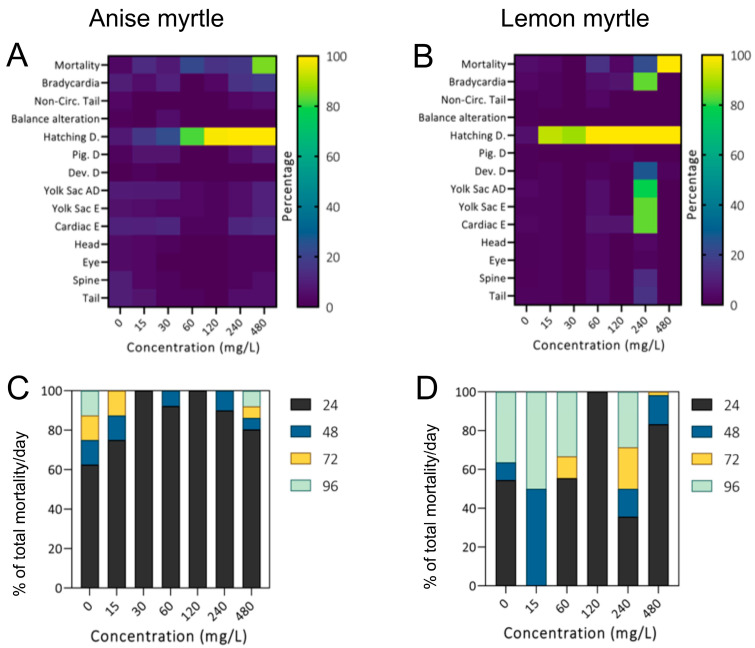
Alterations were observed in zebrafish embryos exposed to anise myrtle (**A**,**C**) and lemon myrtle (**B,D**) extract for 96 h. Heatmap showing the cumulative morbidity and phenotypic and behavioural alterations (**A**,**B**). Hatching delay occurred at higher concentrations for both plant extracts, with other mild alterations. The proportion of total mortality observed each experimental day at different concentrations (**C**,**D**). Colours represent the experimental time in hours (24–96 h). Most of the mortality occurred in the first 24 h, though continued exposure to lemon myrtle showed ongoing mortality continuing by subsequent days.

For all experiments, zebrafish embryos from the control group (0 mg/L) displayed normal morphology and low mortality <5% over the complete experiment. The LC_50_-96h was calculated considering the cumulative percentage of the following signs of mortality: egg coagulation (from 0–24 h of exposure), dead embryos (before hatching, 24–48 h of exposure), and dead larvae (after hatching, 48–96 h of exposure). For anise myrtle extract, the LC_50_-96h calculated was 283.5 mg/L and mortality occurred mainly during the first 24 h of exposure (Figure 1A) for all the concentrations tested. For lemon myrtle extract, the LC_50_-96h calculated was 270.2 mg/L and mortality occurred mainly during the first 24 h of exposure (Figure 1B) for all the concentrations tested, except for 15 mg/L in which the main mortality was observed at 48 and 96 h of exposure. For embryos exposed to 60 and 240 mg/L, substantial mortality was also observed at 96 h, as well as 72 h. Compared to anise myrtle, additional mortality with increasing exposure time was observed in the lemon myrtle toxicity tests.

Exposure to either myrtle resulted primarily in hatching delay across most of the concentrations. In anise myrtle, other alterations in the surviving larvae were very mild, while for lemon myrtle, increased alterations were observed at a concentration of 240 mg/L. The reduced observed alterations at 480 mg/L are likely due to the death of more larvae with those surviving representing those with minimal alterations.

**Figure 2:**
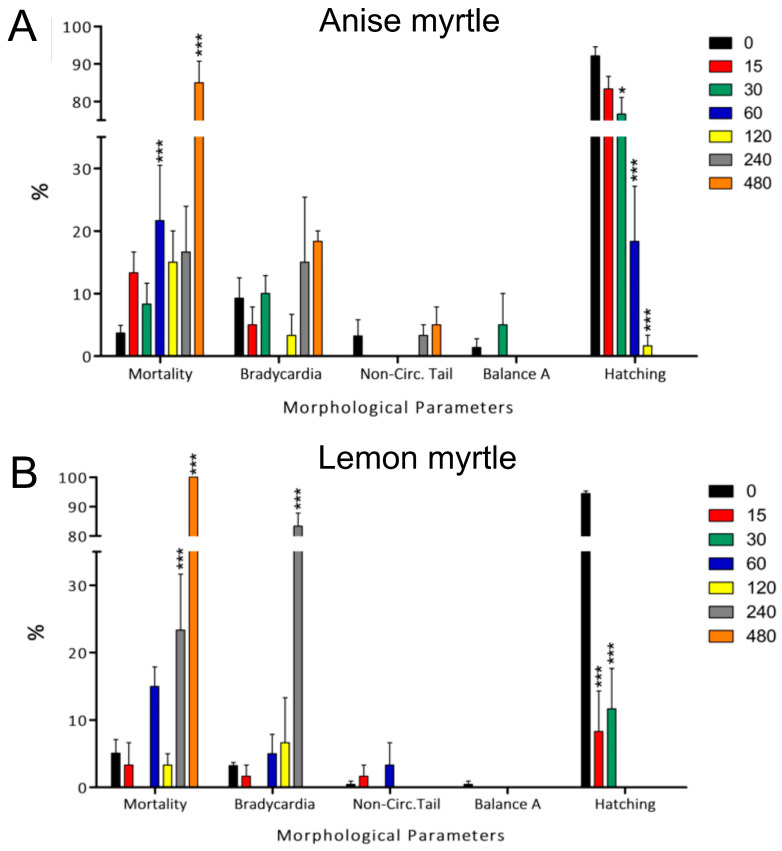
Morphological alterations were observed in zebrafish embryos exposed to anise (**A**) and lemon (**B**) myrtle extract for 96 h and graphed as a percentage of all larvae. The colours represent different concentrations tested (mg/L). Data show the mean ± SD, *n* = 60. Asterisks indicate statistical significance when compared to a control group (0 mg/L): *p* < 0.05 (*) and *p* < 0.001 (***).

Mortality upon anise myrtle exposure was statistically significant (*p* < 0.001) just for the zebrafish larvae exposed to 60 and 480 mg/L (Figure 2A), indicating no concentration-response. In contrast, Lemon myrtle’s mortality was significantly higher at the two highest concentrations (Figure 2B), consistent with a dose response.

Hatching delay was the main morphological trait observed (Figure 2). In general, for both myrtles, balance disorder and signs of morbidity such as bradycardia and loss of blood circulation in the tail were relatively scarce in the surviving larvae, with only bradycardia at 240 mg/L (*p* < 0.001).

**Figure 3:**
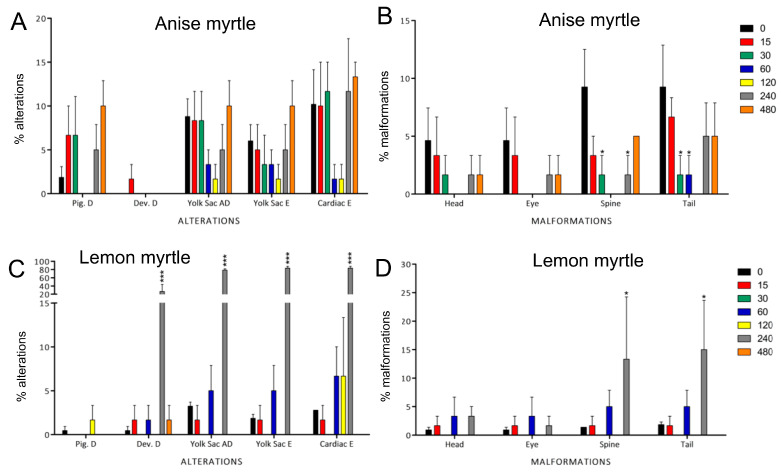
Developmental alterations (**A**,**C**) and malformations (**B**,**D**) were observed in zebrafish larvae exposed at 96 h post-fertilization to different concentrations of anise (**A**,**B**) and lemon (**C**,**D**) myrtle extract. The alterations observed were pigmentation delay (Pig. D), developmental delay (Dev. D), yolk sac absorption delay (Yolk Sac AD), yolk sac edema (Yolk Sac E), and cardiac edema (Cardiac E). The malformations observed were localized in the head, eyes, spine, and tail. None of the alternations observed in anise myrtle were significantly different from the control 0 mg/L group. In contrast, in Lemon myrtle, there were substantial alterations and malformations at 240 mg/L. The 480 mg/L group only includes very few survivors. Bar colours represent the concentrations tested in mg/L. Data represent the mean ± SD, *n* = 60. Asterisks indicate statistical significance when comparing the exposed groups with the control groups: *p* < 0.05 (*) and *p* < 0.001 (***).

In the surviving larvae exposed to anise myrtle, alterations and malformations were relatively limited (Figure 3A,B). Only spine and tail alternations showed a significant but relatively small increase in frequency at some concentrations with a high overall variability. In contrast, surviving larvae exposed to lemon myrtle showed a sudden increase in abnormalities at 240 mg/L. Zebrafish in this exposure group exhibited significant alterations (*p* < 0.001), with developmental delay, yolk sac absorption delay, yolk sac edema, cardiac edema, and malformations (in the spine and tail, *p* < 0.05).

The LC_50_-96h of anise myrtle (283.5 mg/L) was slightly higher than the LC_50_-96h of lemon myrtle (270.2 mg/L). However, lemon myrtle extract caused a higher occurrence of deleterious effects, with increased signs of mortality, morbidity, alterations, and malformations than the embryos exposed to anise myrtle.

## 4. Discussion

For decades, plant-derived bioactive compounds, including alkaloids, polyphenols, and terpenoids, have been recognized for their beneficial effects on health, aiding in the prevention and treatment of Alzheimer’s disease, cancers, obesity, and cardiovascular diseases. Australia is home to unique medicinal plants, among which anise and lemon myrtles are known in traditional medicine for their therapeutic benefits [12]. These plants are rich in antioxidants and exhibit numerous biological properties, including antioxidant, anti-inflammatory, and anti-microbial properties. However, while there have been studies on cell cultures [17,20,39,40], there has been limited research regarding their safety using in vivo studies [41,42]. This study pioneers identifying and analyzing the phytochemicals present in these Australian myrtles and investigates their potential health risk in the in vivo zebrafish model. It is crucial to demonstrate the safety of these natural phytochemicals before considering their use as food additives or in pharmaceuticals.

Our in vivo tests showed that despite the antioxidant activity (Table 1) and abundance of phenolic compounds such as epicatechin and catechin in anise and lemon myrtles (Table 2), respectively, significant adverse effects were caused in zebrafish embryos (Figure 1, Figure 2 and Figure 3). One study revealed that while individual exposure to the five catechins did not induce malformations in zebrafish, embryos exposed to green tea containing catechin exhibited disruptions in dorsoventral differentiation and inhibited epiboly progression [43]. In particular, the constituents present in green tea exhibited a similar effect on embryos as the green tea solution itself. Alafiatayo and colleagues [44] investigated the embryotoxic and teratogenic effects of the methanolic extract of curcuma, a rhizome plant, on zebrafish. Notably, this plant is abundant in flavonoids (catechin and epicatechin), similar to lemon and anise myrtle extracts. The results showed toxic effects and physical deformities in zebrafish larvae exposed to curcuma extract manifested at concentrations above 62.50 μg/mL, which is considerably lower than the concentration (240 mg/L) at which significant toxic effects and malformations were observed in our study involving lemon and anise myrtle extracts. Consistent with our observations, similar teratogenic effects, such as tail and spine malformations, and yolk sac edema, were observed in embryos exposed to the curcuma extract as well as the anise and lemon myrtle extracts. Soares de Araújo Pinho et al. (2014) conducted a study on *Drosophila melanogaster* to evaluate the toxicity of *Duguetia furfuracea* a medicinal plant in Brazil [45]. Despite the antioxidant content present in the hydroalcoholic leaf extracts of *D. furfuracea*, the results revealed adverse effects in the flies, which may be attributed to either the individual or synergistic action of the phytochemicals found in this plant throughout exposure. Other in vivo toxicological studies carried out in zebrafish model include quandong peach (LC_50_ > 480mg/L), Kakadu plum (LC_50_ > 480 mg/L), Davidson plum (LC_50_ 376 mg/L) and muntries (LC_50_ 169 mg/L) [27], pomegranate (LC_50_ 196 mg/L) [46], safflower (LC_50_ 10–60 mg/L at different ages) [47], *Euphorbia kansui* (LC_50_ 345.6 mg/L) [48], Chinese motherwort (*Leonurus japonicus*) (LC_50_ 10 mg/L) [49], *Andrographis paniculata* (LC_50_ 530 mg/L), *Curcuma xanthorrhiza* (LC_50_ 700 mg/L), *Cinnamon zylanicum* (LC_50_ 50 mg/L), *Eugenia polyantha*) (LC_50_ 60 mg/L) and *Orthosiphon stanineus* (LC_50_ 169 mg/L) [50]. While most of the LC_50_ are within the same order of magnitude (most between 60–700 mg/L), differences in extraction methods do not allow direct comparison. The complex biochemical profile of such plants makes it difficult to ascribe the toxicology effects to specific metabolites. The diversity of phytochemicals within species depends on various factors, including the environment, genotype, plant tissue and age, and post-harvest processing and storage. Moreover, extraction conditions and methods, including the temperature, time, type and concentration of solvent, and extraction type, also affect the diversity of phytochemicals [15], which is why toxicology must be correlated to a specific extraction sample.

As more studies are performed on the zebrafish, representing the highest-throughput vertebrate in vivo toxicology model, we can perform standardized analysis for quantitative comparison. Ultimately, correlating toxicity with specific metabolites will allow for improved targeted approaches to selecting potential botanicals. A detailed analysis of the individual components present in these medicinal plants should be undertaken to ascertain their safety profile concerning embryonic development and potential adverse effects on embryos.

The findings of our study thus align with previous research on other medicinal plants, suggesting that the adverse effects observed on zebrafish embryonic development upon exposure to lemon and anise myrtle extracts could be primarily attributed to the cumulative impact of the multiple components present in the extract solutions tested. These findings underscore the significance of conducting thorough toxicological evaluations involving understudied plant products for drug discovery. Furthermore, the results collectively demonstrate that plants with therapeutic potential could also pose potential threats when consumed at higher doses, particularly to embryonic development. Notably, this study represents the first comprehensive assessment of the in vivo toxicity of hydroethanolic extracts derived from anise and lemon myrtle plants.

Phytochemicals identified in our (Table S1) and previous myrtle extracts [15] are found throughout plants and exhibit diverse structures, and molecular weights. Phenolics are recognized for their physiological effects, including antioxidant, anticancer, and antibacterial capabilities, attributed to the presence of phenolic hydroxyl groups. Phytochemical substances have shown efficacy in preventing chronic cardiovascular disease. Specifically, flavonoid compounds that contain hydroxyl groups are responsible for the radical scavenging actions observed in plants. Additional investigation is required to examine the molecular-level antioxidant activity mechanisms of potent compounds, including catechin, epicatechin, ellagic acid arabinoside, isovitexin, procyanidin B2, and myricetin 3-arabinoside extracted from these myrtles.

The phytochemicals in our myrtle leave extracts have been described as possessing potent antioxidant, anti-inflammatory, antimicrobial, antiviral, antifungal and prebiotic capacity, and particularly high levels of phenolics [14,15,40,51,52,53]. Of note, many previous studies have demonstrated beneficial potential from analysis of specifically the essential oils derived from the leaves of the two myrtles, which contain distinct bioactives. Essential oils from both myrtles contain high levels of neral (α-citral) and geranial (β-citral) with high antibacterial activity [10,15,20,40,53,54,55,56,57,58]. In vitro testing of lemon myrtle demonstrated effective anti-inflammatory properties in murine macrophage and human cells [14,15,17,51]. However, despite the great potential of lemon myrtle essential oil as an antimicrobial and antioxidant agent, it has been reported as toxic for mammalian skin cells [20,59], human cell lines (HepG2, F1-73), and primary cell cultures of human skin fibroblasts [20]. Thus, the biological effects of depend closely on the concentration and composition of the extract [12] and thus cannot be correlated across different samples.

The results and effects observed in our current study support the idea that compounds from anise myrtle may be valuable for future pharmaceutical applications. However, their adverse effects need to be studied further in other in vivo models. Anise and lemon myrtle hydroethanolic extracts exhibited similar lethal concentrations on zebrafish embryos (LC_50_-anise: 283.5 mg/L, LC_50_-lemon: 270.2 mg/L). Despite being plants from the same family (Myrtaceae), anise myrtle and lemon myrtle extracts displayed different effects on traits such as bradycardia, developmental delay, yolk sac absorption delay, yolk sac edema, and malformations in the spine and tail. For instance, lemon myrtle displayed a higher percentage of embryos with bradycardia, alterations, and malformations than anise myrtle (Figure 2 and Figure 3). The different effects observed between these plant extracts could be explained by the independent and synergistic effects of specific compounds from each extract, as reported in Table S1. The potent antioxidant properties and rich presence of bioactive compounds in anise and lemon myrtle have motivated further investigations to gain a deeper understanding of the mechanisms underlying the action of these extracts. This knowledge could pave the way for their utilization as novel therapeutic interventions for a wide spectrum of diseases associated with oxidative stress.

## 5. Conclusions

Wild plants, including the incredible diversity of native Australian plants, are still understudied and underutilized for therapeutic approaches. This is despite the critical role of native plants in drug discovery and the unique Australian flora. We identified various bioactive metabolites in both myrtle plant extracts. The differences in the combination and abundance of antioxidant compounds will potentially have distinct benefits against different pathologies. Thus, continued systematic comparison of antioxidant activity and toxicity will help prioritize target plants and compound combinations for further development as drugs.

## Figures and Tables

**Table 1:** Measurement of phenolic contents and their antioxidant activities in anise myrtle and lemon myrtle.

Samples	TPC (mg GAE/g)	DPPH (mM TE/g)	ABTS (mM TE/g)	RPA (mM TE/g)
Anise myrtle	36.8 ± 0.9 ^b^	362.3 ± 6.3 ^b^	415.9 ± 23.8 ^b^	399.8 ± 17.6 ^b^
Lemon myrtle	52.0 ± 2.9 ^a^	421.9 ± 5.2 ^a^	597.1 ± 29.2 ^a^	433.6 ± 19.3 ^a^

The results (mean ± standard deviation, *n* = 3) per gram of sample. TPC: total phenolic content. DPPH: 2,2′-diphenyl-1-picrylhydrazyl assay. ABTS: 2,2′-azino-bis-3-ethylbenzothiazoline-6-sulfonic acid assay. RPA: reducing power assay. GAE: gallic acid equivalent. TE: Trolox equivalent. The results in each column with superscripts (^a,b^) are significantly different (*p*-value < 0.05).

**Table 2:** HPLC-MS quantification/semi-quantification of abundant phenolic metabolites in anise and lemon myrtle extracts.

Compounds	Formula	Anise Myrtle (μg/g)	Lemon Myrtle (μg/g)
Gallic acid	C_7_H_6_O_5_	128.79 ± 5.58 ^b^	232.21 ± 5.10 ^a^
*p*-Hydroxybenzoic acid	C_7_H_6_O_3_	13.08 ± 3.09 ^b^	80.08 ± 12.57 ^a^
Cinnamic acid	C_9_H_8_O_2_	614.12 ± 3.01 ^a^	374.44 ± 30.75 ^b^
Ferulic acid	C_10_H_10_O_4_	3.70 ± 1.25 ^b^	119.87 ± 3.99 ^a^
Syringic acid	C_9_H_10_O_5_	1290.47 ± 27.22	ND
3,4-*O*-dimethygallic acid	C_9_H_10_O_5_	ND	281.83 ± 18.39
Caffeic acid	C_9_H_8_O_4_	184.64 ± 8.41	ND
Caftaric acid	C_13_H_12_O_9_	164.84 ± 11.50	ND
Epicatechin	C_15_H_14_O_6_	4656.04 ± 230.92 ^a^	3821.53 ± 135.11 ^b^
(-)-Epicatechin 3-*O*-gallate	C_22_H_18_O_10_	120.46 ± 3.80 ^b^	1475.81 ± 113.65 ^a^
Isorhamnetin	C_16_H_12_O_7_	164.51 ± 8.99 ^a^	242.43 ± 24.54 ^a^
Kaempferol	C_15_H_10_O_6_	33.52 ± 3.04 ^a^	17.70 ± 1.31 ^b^
Quercetin	C_15_H_10_O_7_	400.03 ± 23.57 ^a^	206.61 ± 20.57 ^b^
Procyanidin B2	C_30_H_26_O_12_	401.33 ± 28.92	1281.58 ± 69.21 ^a^
Pyrogallol	C_6_H_6_O_3_	230.88 ± 3.52 ^b^	423.16 ± 33.01 ^a^
Ellagic acid arabinoside	C_19_H_14_O_12_	2537.93 ± 402.42	ND
*p*-Coumaric acid 4-*O*-glucoside	C_15_H_18_O_8_	116.28 ± 4.72 ^b^	132.26 ± 10.70 ^a^
Isovitexin	C_21_H_20_O_10_	3467.56 ± 270.14 ^a^	403.24 ± 18.87 ^b^
Ellagic acid glucoside	C_10_H_16_O_13_	835.70 ± 59.03	ND
Myricetin 3-arabinoside	C_20_H_18_O_12_	1185.34 ± 74.68	ND
Resveratrol	C_14_H_12_O_3_	ND	116.92 ± 1.10
Sinapic acid	C_11_H_12_O_5_	ND	214.29 ± 3.32
Catechin	C_15_H_14_O_6_	ND	5903.02 ± 762.73
Epigallocatechin	C_15_H_14_O_7_	ND	116.54 ± 3.20

ND: not detected. The results in each column with superscripts (^a,b^) are significantly different from each other (*p*-value < 0.05).

## Data Availability

All data provided in the manuscript.

## Supplementary Materials

The following supporting information can be downloaded at: https://www.mdpi.com/article/10.3390/antiox13080977/s1. Supplementary data include the chromatograms of the myrtles and a Table showing the identification and characterization of phytochemicals from the myrtles.
