# Supplementary material for: Phytochemical Composition and Toxicological Screening of Anise Myrtle and Lemon Myrtle Using Zebrafish Larvae
Source: Antioxidants (Basel). 2024 Aug 12;13(8):977. doi: 10.3390/antiox13080977 (PMC11351381; doi:10.3390/antiox13080977)
Supplement: Supplementary file 1 [file antioxidants-13-00977-s001.zip › antioxidants-3106061-supplementary.pdf]

Supplementary Material

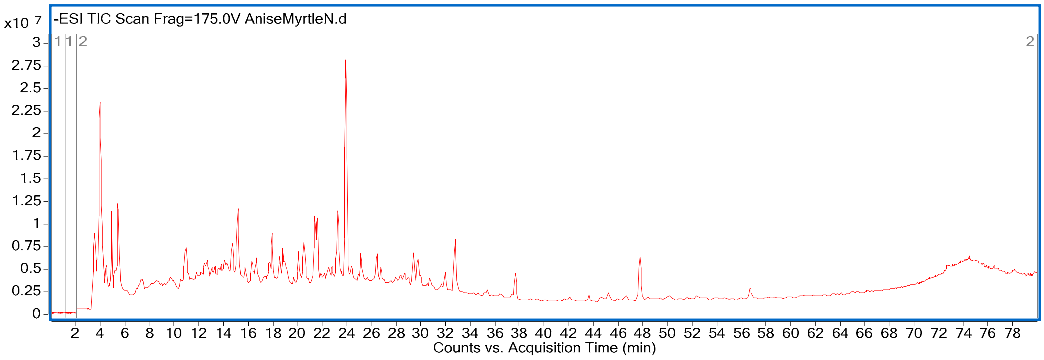

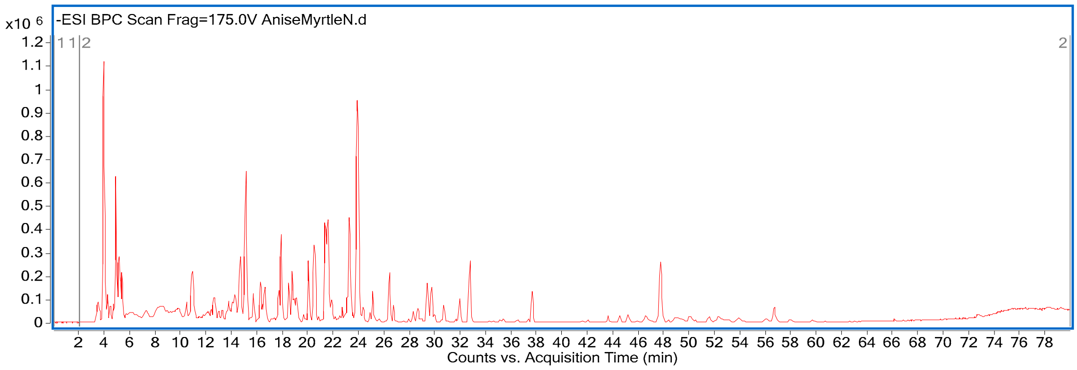

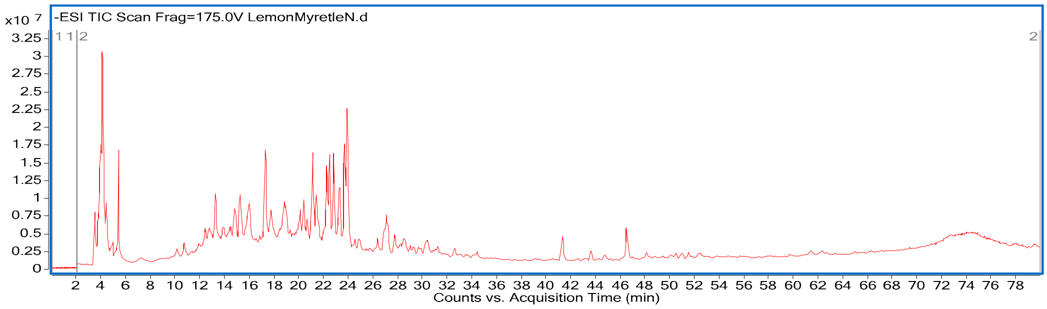

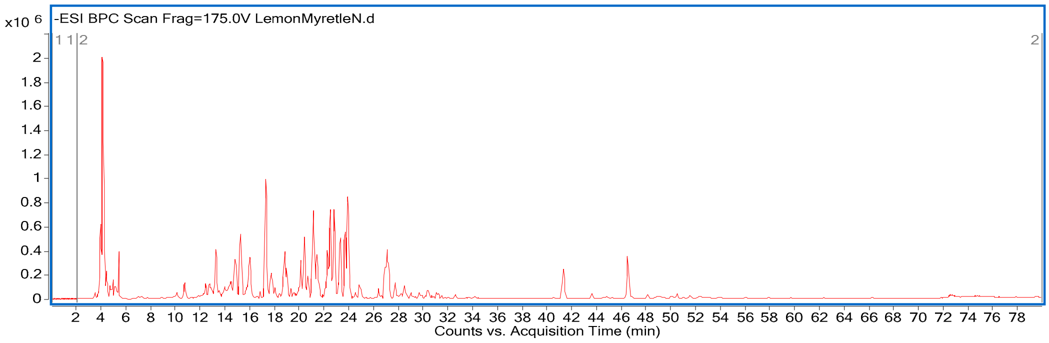

Figure S1. Total Ion Chromatograms (TIC) and Base Peak Chromatograms (BPC) of Lemon Myrtle and Aniseed Myrtle in Negative mode.

Table S1. LC-ESI-QTOF-MS/MS Identification and Characterization of Phytochemicals from Aniseed Myrtle and Lemon Myrtle

No.	Proposed compounds	Molecular Formula	RT (min)	Molecular Weight	Theoretical (*m/z*)	Observed (*m/z*)	Mass Error (ppm)	MS^2	Samples
	**Hydroxybenzoic acids and derivatives**								
1	Gallic acid 4-*O*-glucoside	$C_{13}H_{16}O_{10}$	5.384	332.0744	331.0671	331.0689	5.4	169, 125	AM, LM
2	3-Galloylquinic acid	$C_{14}H_{16}O_{10}$	5.428	344.0744	343.0671	343.0668	-0.9	191, 169, 125	LM
3	Protocatechuic acid 4-*O*-glucoside	$C_{13}H_{16}O_9$	11.151	316.0794	315.0721	315.0720	-0.3	153	AM, LM
4	Valoneic acid dilactone	$C_{21}H_{10}O_{13}$	11.767	470.0121	469.0048	469.0065	3.6	469	AM
5	4-Hydroxybenzoic acid 4-*O*-glucoside	$C_{13}H_{16}O_8$	13.455	300.0845	299.0772	299.0780	2.7	137	AM, LM
6	*p*-Hydroxybenzoic acid	$C_7H_6O_3$	15.268	138.0317	137.0244	137.0240	-2.9	93	LM, AM
7	Ellagic acid glucoside	$C_{20}H_{16}O_{13}$	16.454	464.0591	463.0518	463.0516	-0.4	301	AM
8	Ellagic acid	$C_{14}H_6O_8$	16.629	302.0063	300.9990	300.9986	-1.3	283, 229, 185	AM, LM
9	Benzoic acid	$C_7H_6O_2$	18.932	122.0368	121.0295	121.0295	0.0	105, 93, 77	AM
10	3,4-*O*-Dimethylgallic acid	$C_9H_{10}O_5$	21.844	198.0528	197.0455	197.0447	-4.1	169, 151, 125	LM
11	Gallic acid	$C_7H_6O_5$	22.262	170.0215	169.0142	169.0139	-1.8	125	LM, AM
12	Ellagic acid arabinoside	$C_{19}H_{14}O_{12}$	23.536	434.0485	433.0412	433.0414	0.5	301	AM
	**Hydroxycinnamic acids and derivatives**								
13	Feruloyl tartaric acid	$C_{14}H_{14}O_9$	10.220	326.0638	325.0565	325.0555	-3.1	193, 149, 105	LM
14	Caffeic acid	$C_9H_8O_4$	13.277	180.0423	179.0350	179.0351	0.6	135	AM
15	*p*-Coumaroyl glycolic acid	$C_{11}H_{10}O_5$	13.277	222.0528	221.0455	221.0445	-4.5	177, 145, 119	LM
16	1-*O*-Sinapoyl-β-D-glucose	$C_{17}H_{22}O_{10}$	15.031	386.1213	385.1140	385.1140	0.0	223, 205, 197, 161	AM, LM
17	*p*-Coumaric acid	$C_9H_8O_3$	15.148	164.0473	163.0400	163.0403	1.8	119	AM, LM
18	*p*-Coumaric acid 4-*O*-glucoside	$C_{15}H_{18}O_8$	15.890	326.1002	325.0929	325.0929	0.0	163	AM, LM
19	cis-*p*-Coumaric acid 4-[apiosyl-(1->2)-glucoside]	$C_{20}H_{26}O_{12}$	16.833	458.1424	457.1351	457.1344	-1.5	457	AM, LM
20	Syringic acid	$C_9H_{10}O_5$	17.118	198.0528	197.0455	197.0459	2.0		AM
21	Ferulic acid 4-*O*-glucoside	$C_{16}H_{20}O_9$	17.686	356.1107	355.1034	355.1031	-0.8	193	AM
22	3-Caffeoylquinic acid	$C_{16}H_{18}O_9$	18.770	354.0951	353.0878	353.0884	1.7	191, 179, 161	AM
23	Sinapic acid	$C_{11}H_{12}O_5$	18.960	224.0685	223.0612	223.0618	2.8	193	LM
24	Caftaric acid	$C_{13}H_{12}O_9$	19.541	312.0481	311.0408	311.0409	0.3	249, 161, 135	AM
25	Ferulic acid 4-*O*-glucuronide	$C_{16}H_{18}O_{10}$	21.665	370.0900	369.0827	369.0823	-1.1	193	LM
26	Ferulic acid	$C_{10}H_{10}O_4$	23.432	194.0579	193.0506	193.0497	-4.7	178, 175, 149, 134	AM, LM
27	3-Feruloylquinic acid	$C_{17}H_{20}O_9$	25.002	368.1107	367.1034	367.1018	-4.4	193, 191	AM, LM
28	1,5-Dicaffeoylquinic acid	$C_{25}H_{24}O_{12}$	25.352	516.1268	515.1195	515.1223	5.4	471, 353, 179, 161	LM
29	3-*p*-Coumaroylquinic acid	$C_{16}H_{18}O_8$	28.025	338.1002	337.0929	337.0924	-1.5	191, 163, 119	AM
30	Cinnamic acid	$C_9H_8O_2$	28.095	148.0524	147.0451	147.0443	-5.4	103	AM
32	1-Caffeoyl-5-feruloylquinic acid	$C_{26}H_{26}O_{12}$	30.360	530.1424	529.1351	529.1347	-0.8	193, 191, 179	LM
33	Prenyl cis-caffeic acid	$C_{45}H_{74}O_{19}$	42.464	918.4824	917.4751	917.4739	-1.3	179	LM

	**Other phenolic acids**								
34	2-Hydroxyphenylacetic acid	$C_8H_8O_3$	15.148	152.0473	151.0400	151.0408	5.3	133, 107	AM, LM
35	5-(3'-Methoxy-4'-hydroxyphenyl)-γ-valerolactone	$C_{12}H_{14}O_4$	15.148	222.0892	221.0819	221.0828	4.1	203, 151	AM, LM
36	Dihydrocaffeic acid 3-*O*-glucuronide	$C_{15}H_{18}O_{10}$	15.462	358.0900	357.0827	357.0831	1.1	339, 181	AM
37	3,4-Dihydroxyphenylacetic acid	$C_8H_8O_4$	17.314	168.0423	167.0350	167.0338	-7.2	123	LM
38	3-Phenylpropionic acid	$C_9H_{10}O_2$	23.423	150.0681	149.0608	149.0606	-1.3	105	LM
39	3-Hydroxyphenylvaleric acid	$C_{11}H_{14}O_3$	28.879	194.0943	193.0870	193.0858	-6.2	175, 149	AM
	**Flavanols**								
40	Prodelphinidin trimer GC-GC-C	$C_{45}H_{38}O_{20}$	5.331	898.1956	897.1883	897.1839	-4.9		AM
41	(-)-Epigallocatechin	$C_{15}H_{14}O_7$	10.616	306.0740	305.0667	305.0680	4.3	269, 219	AM, LM
42	Prodelphinidin trimer C-GC-C	$C_{45}H_{38}O_{19}$	12.195	882.2007	881.1934	881.1925	-1.0		AM, LM
43	Procyanidin B2	$C_{30}H_{26}O_{12}$	14.104	578.1424	577.1351	577.1359	1.4	289, 245	AM, LM
44	Procyanidin trimer C1	$C_{45}H_{38}O_{18}$	14.548	866.2058	865.1985	865.1969	-1.8	245	AM, LM
45	(+)-Catechin	$C_{15}H_{14}O_6$	15.148	290.0790	289.0717	289.0728	3.8	245	LM
46	Epicatechin	$C_{15}H_{14}O_6$	17.605	290.0790	289.0717	289.0728	3.8	245	AM, LM
47	Cinnamtannin A2	$C_{60}H_{50}O_{24}$	17.808	1154.2692	1153.2619	1153.2608	-1.0		AM
48	(+)-Gallocatechin 3-*O*-gallate	$C_{22}H_{18}O_{11}$	18.069	458.0849	457.0776	457.0733	-9.4	305, 169	LM
49	(-)-Epicatechin 3-*O*-gallate	$C_{22}H_{18}O_{10}$	19.867	442.0900	441.0827	441.0829	0.5	289, 245, 169	LM
50	(-)-Epigallocatechin 7-*O*-glucuronide	$C_{21}H_{22}O_{13}$	19.867	482.1060	481.0987	481.0981	-1.2	305, 169	LM
51	3'-*O*-Methylcatechin	$C_{16}H_{16}O_6$	20.965	304.0947	303.0874	303.0857	-5.6	289, 245	LM
52	Theaflavin 3,3'-*O*-digallate	$C_{43}H_{32}O_{20}$	23.273	868.1487	867.1414	867.1495	9.3	715, 563, 169, 125	AM
53	Theaflavin	$C_{29}H_{24}O_{12}$	28.046	564.1268	563.1195	563.1192	-0.5	441, 163	LM
54	Prodelphinidin dimer B3	$C_{30}H_{26}O_{14}$	28.357	610.1323	609.1250	609.1249	-0.2	591, 539	LM, AM
	**Flavonols**								
55	Isorhamnetin 3-*O*-glucoside 7-*O*-rhamnoside	$C_{28}H_{32}O_{16}$	15.079	624.1690	623.1617	623.1607	-1.6	315	AM
56	Myricetin 3-*O*-rutinoside	$C_{27}H_{30}O_{17}$	15.462	626.1483	625.1410	625.1396	-2.2	317	AM
57	Kaempferol 3-*O*-xylosyl-glucoside	$C_{26}H_{28}O_{15}$	15.997	580.1428	579.1355	579.1367	2.1	285	LM
58	Quercetin 4'-*O*-glucuronide	$C_{21}H_{18}O_{13}$	17.151	478.0747	477.0674	477.0673	-0.2	301	LM
59	3-Methoxysinensetin	$C_{21}H_{22}O_8$	17.321	402.1315	401.1242	401.1241	-0.2	401	AM
60	Europetin 3-galactoside	$C_{22}H_{22}O_{13}$	17.391	494.1060	493.0987	493.0992	1.0	331	LM
61	6-Hydroxyquercetin	$C_{15}H_{10}O_8$	18.537	318.0376	317.0303	317.0300	-0.9	299, 151	AM, LM
62	Kaempferol 3-*O*-glucuronide	$C_{21}H_{18}O_{12}$	18.537	462.0798	461.0725	461.0741	3.5	285	AM
63	Kaempferol 3,7-*O*-diglucoside	$C_{27}H_{30}O_{16}$	19.088	610.1534	609.1461	609.1453	-1.3	285	AM
64	Myricetin 3-*O*-arabinoside	$C_{20}H_{18}O_{12}$	19.759	450.0798	449.0725	449.0727	0.4	317	AM, LM
65	Quercetin 3-*O*-glucosyl-xyloside	$C_{26}H_{28}O_{16}$	19.837	596.1377	595.1304	595.1313	1.5	301	AM
66	Dihydromyricetin 3-*O*-rhamnoside	$C_{21}H_{22}O_{12}$	20.219	466.1111	465.1038	465.1041	0.6	319	LM
67	Quercetin 3-*O*-xyloside	$C_{20}H_{18}O_{11}$	23.280	434.0849	433.0776	433.0764	-2.8	301	LM, AM
68	Taxifolin 4',7-diglucoside	$C_{27}H_{32}O_{17}$	23.570	628.1640	627.1567	627.1575	1.3	303	AM

69	3'-O-Methylmyricetin (Annulatin)	$C_{16}H_{12}O_8$	23.717	332.0532	331.0459	331.0468	2.7	317	LM
70	Quercetin 3-*O*-glucoside	$C_{21}H_{20}O_{12}$	24.480	464.0955	463.0882	463.0877	-1.1	301	AM, LM
71	Myricetin 3-*O*-glucoside	$C_{21}H_{20}O_{13}$	25.671	480.0904	479.0831	479.0827	-0.8	317	AM
72	Kaempferol	$C_{15}H_{10}O_6$	26.450	286.0477	285.0404	285.0397	-2.5	285	AM, LM
73	Quercetin	$C_{15}H_{10}O_7$	28.312	302.0426	301.0353	301.0355	0.7	283, 272, 179, 151	AM, LM
74	Isorhamnetin	$C_{16}H_{12}O_7$	45.412	316.0510	315.0510	315.0513	1.0	300, 151, 107	AM, LM
75	5-Hydroxy-3,3',7,8-tetramethoxy-4',5'-methylenedioxyflavone	$C_{20}H_{18}O_9$	37.714	402.0951	401.0878	401.0869	-2.2	209, 151	LM
76	3,7-Dimethylquercetin	$C_{17}H_{14}O_7$	46.524	330.0740	329.0667	329.0640	-8.2	299, 283	LM
	**Flavones**								
77	Lilaline	$C_{20}H_{17}NO_7$	3.979	383.1005	382.0932	382.0942	2.6	382	AM
78	Multijugin	$C_{24}H_{22}O_7$	5.293	422.1366	421.1293	421.1311	4.3	421	LM
79	Tephrodin	$C_{25}H_{22}O_8$	8.259	450.1315	449.1242	449.1271	6.5	449	LM
80	Heterophynone	$C_{27}H_{34}O_6$	12.043	454.2355	453.2282	453.2280	-0.4	453	LM
81	Artonin V	$C_{25}H_{26}O_7$	13.345	438.1678	437.1605	437.1604	-0.2	437	AM
82	5,7-Dihydroxyflavone 7-benzoate	$C_{22}H_{14}O_5$	15.554	358.0841	357.0768	357.0770	0.6	357	LM
83	Artonin E	$C_{25}H_{24}O_7$	16.780	436.1522	435.1449	435.1424	-5.7	357	AM
84	Dalspinin	$C_{17}H_{12}O_7$	17.923	328.0583	327.0510	327.0512	0.6	327	AM
85	Pongamoside A	$C_{23}H_{20}O_9$	18.017	440.1107	439.1034	439.1033	-0.2	439	LM
86	Pratensin A	$C_{23}H_{22}O_9$	20.262	442.1264	441.1191	441.1189	-0.5	441	LM
87	Quercetin 3-(2-galloylglucoside)	$C_{28}H_{24}O_{16}$	20.346	616.1064	615.0991	615.0987	-0.7	301	AM, LM
88	Exoticin	$C_{23}H_{26}O_{10}$	21.279	462.1526	461.1453	461.1466	2.8	461	AM
89	Tricin 7-*O*-glucuronide	$C_{23}H_{22}O_{13}$	22.794	506.1060	505.0987	505.0995	1.6	329	AM, LM
90	Syringetin-3-*O*-glucoside	$C_{23}H_{24}O_{13}$	23.280	508.1217	507.1144	507.1151	1.4	345	LM
91	Kaempferol 3-triglucoside 7-rhamnoside	$C_{39}H_{50}O_{25}$	23.280	918.2641	917.2568	917.2620	5.7	285	LM
92	6-Hydroxyluteolin 7-*O*-rhamnoside	$C_{21}H_{20}O_{11}$	23.913	448.1006	447.0933	447.0943	2.2	447	LM, AM
93	Chrysoeriol 7-*O*-glucoside	$C_{22}H_{22}O_{11}$	24.369	462.1162	461.1089	461.1094	1.1	461	AM, LM
94	Apigenin 7-*O*-glucuronide	$C_{21}H_{18}O_{11}$	24.930	446.0849	445.0776	445.0760	-3.6	269	AM
95	Apigenin 6-C-glucoside	$C_{21}H_{20}O_{10}$	26.450	432.1056	431.0983	431.0984	0.2	269	AM, LM
96	3,5-Dimethylquercetin glucoside	$C_{23}H_{24}O_{12}$	26.504	492.1268	491.1195	491.1195	0.0	491	AM, LM
97	4',5,7,8-Tetramethoxyflavone	$C_{19}H_{18}O_6$	28.606	342.1103	341.1030	341.1026	-1.2	341	LM
98	Apigenin 6,8-C-arabinoside-C-glucoside	$C_{26}H_{28}O_{14}$	28.719	564.1479	563.1406	563.1400	-1.1	261	LM
99	Swertisin	$C_{22}H_{22}O_{10}$	29.992	446.1213	445.1140	445.1139	-0.2	325, 297, 282	LM
100	Casticin	$C_{19}H_{18}O_8$	30.234	374.1002	373.0929	373.0935	1.6	373	LM
101	Rhoifolin	$C_{27}H_{30}O_{14}$	30.916	578.1636	577.1563	577.1562	-0.2	459, 431, 269, 151	LM
	**Chalcones and dihydrochalcones**								
102	Olivin	$C_{17}H_{16}O_6$	22.615	316.0947	315.0874	315.0867	-2.2	125	AM
103	Phloridzin	$C_{21}H_{24}O_{10}$	24.421	436.1370	435.1297	435.1279	-4.1	273, 167, 123	AM, LM
104	Phloretin 2'-*O*-glucuronide	$C_{21}H_{22}O_{11}$	24.916	450.1162	449.1089	449.1088	-0.2	273	LM
105	6'-Hydroxy-4,2',3',4'-	$C_{19}H_{20}O_6$	27.401	344.1260	343.1187	343.1180	-2.0	328, 298	LM

	tetramethoxychalcone								
106	4,3'-Hydroxy-2',4',5',6'-methoxychalcone	$C_{19}H_{20}O_7$	31.783	360.1209	359.1136	359.1137	0.3		LM
107	Phloretin 2'-*O*-xylosyl-glucoside	$C_{26}H_{32}O_{14}$	31.783	568.1792	567.1719	567.1706	-2.3	273	LM
	**Flavanones**								
108	Naringenin 7-*O*-glucoside	$C_{21}H_{22}O_{10}$	4.055	434.1213	433.1140	433.1132	-1.8	271	AM, LM
109	Poncirin	$C_{28}H_{34}O_{14}$	18.177	594.1949	593.1876	593.1880	0.7	447, 285, 163,	AM
110	Kaempferol 7-(6''-galloylglucoside)	$C_{28}H_{24}O_{15}$	22.503	600.1115	599.1042	599.1036	-1.0	285	AM, LM
111	Hesperetin 3'-*O*-glucuronide	$C_{22}H_{22}O_{12}$	23.717	478.1111	477.1038	477.1042	0.8	301	LM, AM
112	Narirutin	$C_{27}H_{32}O_{14}$	26.218	580.1792	579.1719	579.1745	4.5	271, 151	LM
113	Brosimacutin C	$C_{20}H_{22}O_5$	28.046	342.1467	341.1394	341.1390	-1.2	341	LM
114	Persicogenin 5-glucoside	$C_{23}H_{26}O_{11}$	30.234	478.1475	477.1402	477.1407	1.0	315	LM
115	Muscomin	$C_{18}H_{18}O_7$	40.869	346.1052	345.0979	345.0977	-0.6	345	LM
	**Isoflavonoids**								
116	Luteone 7-glucoside	$C_{26}H_{28}O_{11}$	4.750	516.1632	515.1559	515.1552	-1.4	353	AM
117	Daidzein 7-*O*-glucuronide	$C_{21}H_{18}O_{10}$	17.923	430.0900	429.0827	429.0865	8.9	253	AM
118	3'-Hydroxymelanettin	$C_{16}H_{12}O_6$	18.770	300.0634	299.0561	299.0563	0.7	299	AM
119	Irisolidone 7-*O*-glucuronide	$C_{23}H_{22}O_{12}$	19.024	490.1111	489.1038	489.1020	-3.7	313	LM
120	2'-Hydroxyformononetin	$C_{16}H_{12}O_5$	20.505	284.0685	283.0612	283.0610	-0.7	283	AM
121	3',4',7-Trihydroxyisoflavanone	$C_{15}H_{12}O_5$	24.381	272.0685	271.0612	271.0607	-1.8	271	LM
122	3'-Hydroxy-*O*-desmethylangolensin	$C_{15}H_{14}O_5$	25.577	274.0841	273.0768	273.0764	-1.5	273	LM
123	Eriodictyol	$C_{15}H_{12}O_6$	32.057	288.0634	287.0561	287.0566	1.7	287	LM
	**Extended flavonoids**								
124	Dihydrocycloartomunin	$C_{26}H_{26}O_7$	4.546	450.1678	449.1605	449.1621	3.6	449	LM
125	Artelastochromene	$C_{30}H_{30}O_6$	28.458	486.2042	485.1969	485.1970	0.2	485	LM
	**Other flavonoids**								
126	Hoslundin	$C_{23}H_{18}O_7$	4.200	406.1052	405.0979	405.0974	-1.2	405	LM
127	7,4'-Dimethoxyflavone	$C_{17}H_{14}O_4$	4.319	282.0892	281.0819	281.0846	9.6	281	LM
128	4'-Chloroaurone	$C_{15}H_9ClO_2$	4.319	256.0291	255.0218	255.0229	4.3	255	LM
129	8-Hydroxyquercetagetin	$C_{15}H_{10}O_9$	4.714	334.0325	333.0252	333.0267	4.5	333	AM
130	(S)-5,7-Dihydroxy-6-prenylflavanone	$C_{20}H_{20}O_4$	5.428	324.1362	323.1289	323.1288	-0.3	323	LM
131	8-*p*-Hydroxybenzylquercetin	$C_{22}H_{16}O_8$	12.778	408.0845	407.0772	407.0761	-2.7	407	LM, AM
132	Dalpalatin	$C_{18}H_{14}O_8$	17.923	358.0689	357.0616	357.0625	2.5	357	AM, LM
133	Cuneatin	$C_{17}H_{12}O_6$	20.505	312.0634	311.0561	311.0568	2.3	311	AM
134	3',4',5'-Trimethoxyflavone	$C_{18}H_{16}O_5$	22.550	312.0998	311.0925	311.0907	-5.8	311	AM
135	Kanzonol L	$C_{30}H_{32}O_6$	26.123	488.2199	487.2126	487.2147	4.3	487	LM
136	Kaempferol 3-(2''-(E)-feruloylgalactosyl-(1->4)-glucoside)	$C_{37}H_{38}O_{19}$	29.066	786.2007	785.1934	785.1908	-3.3	285	LM
137	5-Hydroxyauranetin	$C_{20}H_{20}O_8$	48.141	388.1158	387.1085	387.1059	-6.7	387	LM
138	Cycloheterophyllin	$C_{30}H_{30}O_7$	25.849	502.1992	501.1919	501.1924	1.0	501	LM
139	Hoslunddiol	$C_{22}H_{22}O_7$	43.759	398.1366	397.1293	397.1290	-0.8	397	LM

	**Tannins**								
140	Grandinin	$C_{46}H_{34}O_{30}$	5.434	1066.1135	1065.1062	1065.1041	-2.0	1047	AM
141	Vescalagincarboxylic acid	$C_{42}H_{26}O_{27}$	5.523	962.0662	961.0589	961.0572	-1.8	961	AM
142	2-*O*-Galloylpunicalin	$C_{41}H_{26}O_{26}$	8.486	934.0712	933.0639	933.0634	-0.5	933	AM
143	Punigluconin	$C_{34}H_{26}O_{23}$	8.940	802.0865	801.0792	801.0789	-0.4	801	AM
144	Pedunculagin	$C_{34}H_{24}O_{22}$	10.468	784.0759	783.0686	783.0683	-0.4	783	AM, LM
145	Prodelphinidin B4 3'-gallate	$C_{37}H_{30}O_{18}$	13.039	762.1432	761.1359	761.1316	-5.6	761	LM
146	Camelliatannin E	$C_{49}H_{38}O_{28}$	13.455	1074.1550	1073.1477	1073.1423	-5.0	1073	AM
147	Potentillin	$C_{41}H_{28}O_{26}$	14.716	936.0869	935.0796	935.0786	-1.1	935	AM
148	Punicafolin	$C_{41}H_{30}O_{26}$	19.474	938.1025	937.0952	937.0944	-0.9	937	AM
149	Procyanidin C1 3',3''-digallate	$C_{59}H_{46}O_{26}$	23.999	1170.2277	1169.2204	1169.2180	-2.1	1169	LM
	**Coumarins and derivatives**								
150	7-Methoxycoumarin	$C_{10}H_8O_3$	4.414	176.0473	175.0400	175.0397	-1.7	133	LM
151	Urolithin B 3-*O*-glucuronide	$C_{19}H_{16}O_9$	4.443	388.0794	387.0721	387.0704	-4.4	387	LM
152	Khelmarin D	$C_{28}H_{24}O_8$	12.144	488.1471	487.1398	487.1423	5.1	487	AM
153	5,7-Dimethoxycoumarin	$C_{11}H_{10}O_4$	15.148	206.0579	205.0506	205.0517	5.4	205	AM, LM
154	Scopoletin	$C_{10}H_8O_4$	20.729	192.0423	191.0350	191.0351	0.5	191	LM
155	Urolithin C	$C_{13}H_8O_5$	23.913	244.0372	243.0299	243.0305	2.5	243	LM
156	Urolithin A	$C_{13}H_8O_4$	26.450	228.0423	227.0350	227.0351	0.4	227	AM, LM
157	Esculin	$C_{15}H_{16}O_9$	29.905	340.0794	339.0721	339.0713	-2.4	149, 133, 89	AM, LM
	**Phenolic terpenes**								
158	Rosmadial	$C_{20}H_{24}O_5$	26.398	344.1624	343.1551	343.1540	-3.2	343	LM
159	Carvacrol	$C_{10}H_{14}O$	66.196	150.1045	149.0972	149.0967	-3.4	105	LM
	**Tyrosols**								
160	Oleoside 11-methylester	$C_{17}H_{24}O_{11}$	13.504	404.1319	403.1246	403.1259	3.2	389	AM
161	Oleoside dimethylester	$C_{18}H_{26}O_{11}$	15.348	418.1475	417.1402	417.1415	3.1	389	AM
	**Stilbenes**								
162	Batatasin II	$C_{16}H_{18}O_4$	15.554	274.1205	273.1132	273.1140	2.9	273	LM
163	Resveratrol	$C_{14}H_{12}O_3$	21.032	228.0786	227.0713	227.0710	-1.3	227	LM
164	Resveratrol 3-*O*-glucoside (Polydatin)	$C_{20}H_{22}O_8$	24.381	390.1315	389.1242	389.1229	-3.3	227	LM
165	Piceatannol 3-*O*-glucoside	$C_{20}H_{22}O_9$	26.548	406.1264	405.1191	405.1192	0.2	243	LM
	**Lignans**								
166	Syringaresinol	$C_{22}H_{26}O_8$	21.969	418.1628	417.1555	417.1557	0.5	417	LM
167	Secoisolariciresinol-sesquilignan	$C_{30}H_{38}O_{10}$	25.785	558.2465	557.2392	557.2391	-0.2	557	LM
168	Lariciresinol-sesquilignan	$C_{30}H_{36}O_{10}$	26.398	556.2308	555.2235	555.2212	-4.1	555	LM
169	(8R,8'R)-Secoisolariciresinol 9-glucoside	$C_{26}H_{36}O_{11}$	27.417	524.2258	523.2185	523.2202	3.2	523	AM, LM
170	Trachelogenin	$C_{21}H_{24}O_7$	28.200	388.1522	387.1449	387.1455	1.5	371, 329, 235	AM
171	Sesaminol glucoside	$C_{26}H_{28}O_{12}$	30.540	532.1581	531.1508	531.1470	-7.2	369	AM
	**Other polyphenols**								
172	Amaronol B	$C_{16}H_{14}O_8$	3.686	334.0689	333.0616	333.0598	-5.4	333	AM

173	Norfuraneol	$C_5H_6O_3$	4.081	114.0317	113.0244	113.0245	0.9	113	AM
174	Furaneol	$C_6H_8O_3$	4.160	128.0473	127.0400	127.0392	-6.3	127	LM
175	Quinic Acid	$C_7H_{12}O_6$	14.639	192.0634	191.0561	191.0558	-1.6	127, 93	AM, LM
176	Guaiacol	$C_7H_8O_2$	15.148	124.0524	123.0451	123.0459	6.5	123	AM, LM
177	Pyrogallol	$C_6H_6O_3$	22.262	126.0317	125.0244	125.0244	0.0	107, 97, 79	LM, AM
178	Norathyriol	$C_{13}H_8O_6$	23.717	260.0321	259.0248	259.0255	2.7	169, 139, 97	LM, AM
179	Salvianolic acid G	$C_{20}H_{18}O_{10}$	24.916	418.0900	417.0827	417.0821	-1.4	417	LM, AM
180	Cearoin	$C_{14}H_{12}O_4$	34.064	244.0736	243.0663	243.0652	-4.5	243	LM
	**Limonoids**								
181	Obacunone 17-β-D-glucoside	$C_{32}H_{42}O_{13}$	26.313	634.2625	633.2552	633.2556	0.6	453	AM
182	Isolimonoic acid	$C_{26}H_{34}O_{10}$	31.783	506.2152	505.2079	505.2094	3.0	505	LM
	**Sesquiterpenoids**								
183	Phytuberin	$C_{17}H_{26}O_4$	45.262	294.1831	293.1758	293.1761	1.0	251, 233	AM, LM

AM, Aniseed myrtle; LM, Lemon myrtle
